# Rabies in a Dog Imported from Azerbaijan — Pennsylvania, 2021

**DOI:** 10.15585/mmwr.mm7120a3

**Published:** 2022-05-20

**Authors:** Florence Whitehill, Sarah Bonaparte, Claire Hartloge, Lauren Greenberg, Panayampalli S. Satheshkumar, Lillian Orciari, Michael Niezgoda, Pamela A. Yager, Emily G. Pieracci, Jacquelyn McCullough, Anders Evenson, Clive M. Brown, Hannah Schnitzler, Beth Lipton, Kimberly Signs, Mary Grace Stobierski, Connie Austin, Staci Slager, Mark Ernst, Janna Kerins, Aliza Simeone, Amber Singh, Shelby Hale, Danielle Stanek, Patrick Shehee, Sally Slavinski, Darby McDermott, Patricia A. Zinna, Rebecca Campagna, Ryan M. Wallace

**Affiliations:** ^1^Epidemic Intelligence Service, CDC; ^2^Division of High-Consequence Pathogens and Pathology, National Center for Emerging and Zoonotic Infectious Diseases, CDC; ^3^Division of Global Migration and Quarantine, National Center for Emerging and Zoonotic Infectious Diseases, CDC; ^4^Washington State Department of Health; ^5^Public Health – Seattle & King County, Seattle, Washington; ^6^Michigan Department of Health and Human Services; ^7^Illinois Department of Public Health; ^8^Illinois Department of Agriculture; ^9^Chicago Department of Public Health, Illinois; ^10^Pennsylvania Department of Agriculture; ^11^Ohio Department of Health; ^12^Florida Department of Health; ^13^New York City Department of Health and Mental Hygiene, New York; ^14^New Jersey Department of Health; ^15^California Department of Public Health.

On June 16, 2021, rabies virus infection was confirmed in a dog included in a shipment of rescue animals imported into the United States from Azerbaijan. A multistate investigation was conducted to prevent secondary rabies cases, avoid reintroduction of a dog-maintained rabies virus variant (DMRVV), identify persons who might have been exposed and would be recommended to receive rabies postexposure prophylaxis, and investigate the cause of importation control failures. Results of a prospective serologic monitoring (PSM) protocol suggested that seven of 32 (22%) animals from the same shipment as the dog with confirmed rabies virus infection and who had available titer results after rabies vaccine booster had not been adequately vaccinated against rabies before importation. A requirement for rabies vaccination certificates alone will not adequately identify improper vaccination practices or fraudulent paperwork and are insufficient as a stand-alone rabies importation prevention measure. Serologic titers before importation would mitigate the risk for importing DMRVV.

## Investigation and Findings

On June 10, 2021, a shipment of 33 dogs and one cat arrived at O’Hare International Airport in Chicago, Illinois, from Baku, Azerbaijan, a country designated by CDC as being at high risk for DMRVV.* All 34 animals had valid rabies vaccination certificates and appeared healthy on visual inspection by CDC quarantine station staff members. The shipment appeared to meet entry requirements in place at the time.^†^ The animals were transferred to caretakers and relocated by ground transportation to nine states: California, Florida, Illinois, Michigan, New Jersey, New York, Ohio, Pennsylvania, and Washington.

On June 13, one dog (dog A), a 5-month-old mixed breed male weighing 18.5 lbs (8.4 kg), was hospitalized at a Pennsylvania veterinary clinic because of diarrhea and neurologic changes that began June 12, including biting invisible objects (i.e., fly-biting behavior), hypersalivation, and agitation. While hospitalized, dog A experienced seizure-like activity, obtundation, and sudden cardiac arrest. Clinical signs were consistent with rabies virus infection. After Dog A was stabilized by cardiopulmonary resuscitation and intubation, its owners elected humane euthanasia. Dog A was euthanized on June 13 and submitted to the Pennsylvania Department of Health, Bureau of Laboratories for rabies testing.

On June 16, the Pennsylvania Department of Health Bureau of Laboratories confirmed rabies virus infection by direct fluorescent antibody test. On June 18, CDC Rabies Laboratory confirmed by antigenic variant typing that dog A was infected with a DMRVV. Complete rabies virus nucleoprotein and glycoprotein sequences displayed high identity (approximately 99.5% and 99%, respectively) with a reference rabies virus isolate from a dog in Azerbaijan in 2002.

## Public Health Response

The CDC Poxvirus and Rabies Branch and the Quarantine and Border Health Services Branch led a multistate investigation to implement prevention and control measures and to determine the source of importation control failures. The Chicago Department of Public Health and the Pennsylvania Department of Agriculture investigated potential human exposures to dog A. During June 10–13, dog A traveled by private vehicle to the adopting family’s home, two pet stores, and one veterinary clinic. Thirty-seven persons in the United States had potential exposures to dog A during its infectious period from June 2 (10 days before symptom onset) to June 13. After risk assessments, 15 persons, including one airport worker, seven household contacts, three pet store workers, and four veterinary staff members reported exposures that could have resulted in rabies virus infection and were recommended to receive rabies postexposure prophylaxis.^§^ Three additional persons pursued postexposure prophylaxis out of an abundance of caution. Through the network of national focal points maintained by the World Health Organization under the International Health Regulations, CDC informed the Azerbaijani government and recommended contact tracing.

Antemortem serum specimens from dog A collected on June 13 were tested for rabies virus–neutralizing antibody titer by the rapid fluorescent focus inhibition test at CDC’s Rabies Laboratory. The result (0.3 IU/mL) was less than the cut-off value (0.5 IU/mL) established by the World Organisation for Animal Health (OIE) for adequate neutralizing antibody response to rabies vaccination. This finding could reflect an antibody response from inadequate vaccination or early production of neutralizing antibody in response to rabies virus infection.

Whether or not the other animals in the shipment were in direct contact with dog A during its infectious period is unclear; therefore, all animals in the shipment were considered exposed. Vaccination histories for all other animals in the shipment were considered unreliable after dog A was confirmed to have a rabies virus infection. Exposed animals were evaluated by PSM, a method recognized by the National Association of State Public Health Veterinarians for evaluating the vaccination status of dogs or cats with an uncertain vaccine history (*1*). PSM entails drawing a baseline serum sample, then immediately administering a rabies vaccine booster. A second serum sample is obtained between day 5 and day 7, and titers are determined by rapid fluorescent focus inhibition test. Dogs and cats with a second titer result of ≥0.5 IU/mL and more than a twofold rise in titer compared to baseline are considered to be adequately vaccinated against rabies. Failure to meet these criteria offers evidence of inadequate or absent previous rabies vaccination. CDC developed a protocol to assist states with collecting samples and determining quarantine requirements based on serologic results (Supplementary Box, https://stacks.cdc.gov/view/cdc/116629).

State health departments arranged for the 33 exposed animals (32 dogs and one cat) to receive rabies booster vaccination. Among 30 animals with available prebooster serum samples, 14 (47%) had titers <0.5 IU/mL, indicating that they had inadequate titers at importation. Seven of 32 (22%) animals with available postbooster serum samples failed to meet PSM requirements and were considered previously unvaccinated or inadequately vaccinated. One that did not have available serum samples was treated as previously unvaccinated. These eight animals underwent strict quarantine for 4–6 months, in compliance with state laws. Twenty-five of 32 (78%) animals with paired postbooster serum samples met PSM requirements, were considered previously vaccinated, and were required to undergo a 45-day in-home quarantine because of their presumed rabies exposure. None of the exposed animals died or exhibited rabies symptoms under quarantine. All animals completed the required quarantines by December 29, 2021.

The eight dogs that failed to meet PSM requirements had been housed at the same rescue location in Azerbaijan and were reported to have been vaccinated at the same veterinary clinic in Azerbaijan (clinic A) with a vaccine produced by manufacturer A. Manufacturer A reported no issues with efficacy or variation across the vaccine lots recorded for these animals. Among dogs vaccinated at clinic A, the risk for PSM protocol failure was 3.6 times as high among large dogs (i.e., ≥39.7 lbs [18 kg]) compared with that among smaller dogs (i.e., <39.7 lbs) (95% CI = 0.5–25.6) (Table).

**TABLE:** Relative risk of prospective serologic monitoring failure and geometric mean titer of rabies virus–neutralizing antibody* among 24 dogs^†^^,^^§^ in a shipment of rescue animals imported from Azerbaijan, by vaccine history risk factor — United States, June 2021

Risk factor	Characteristic	No.^†^	Prospective serologic monitoring failure			Geometric mean titer	
			No. (%)	Relative risk (95% CI)	p-value^¶^	Titer (IU/mL)	p-value**
**Vaccine**	Manufacturer A	21	5 (24)	Inf (—)	0.48	2.08	0.29
	Manufacturer B (Ref)	3	0 (—)			7.46	
**Rescue**	Rescue A	16	5 (31)	Inf (—)	0.13	1.87	0.34
	Other (Ref)	8	0 (—)			4.18	
**Veterinary clinic**	Clinic A	19	5 (26)	Inf (—)	0.27	2.33	0.82
	Clinic B (Ref)	5	0 (—)			2.92	
**Animal body weight^††^**	≥39.7 lbs (18 kg)	11	4 (36)	4.0 (0.5–30.3)	0.17	1.46	0.28
	<39.7 lbs (Ref)	11	1 (9)			3.67	
Veterinary clinic A	≥39.7 lbs	9	4 (44)	3.6 (0.5–25.6)	0.24	0.99	0.11
	<39.7 lbs (Ref)	8	1 (13)			5.21	
Veterinary clinic B	≥39.7 lbs	2	0 (—)	—	—	8.33	0.17
	<39.7 lbs (Ref)	3	0 (—)			1.44	

**Abbreviations:** Inf = infinity; Ref = referent group.

* Measured by rapid fluorescent focus inhibition test.

^†^ The shipment also included one cat, which was excluded from analysis.

^§^ Ten dogs were excluded from analysis because of one of the following reasons: known illness, outlier titer result, missing data, or history of multiple vaccinations before import to the United States.

^¶^ Assessed using Fisher’s exact test.

** Assessed using t-test.

^††^ Body weight was not reported for two dogs.

CDC received confirmation from the rescue organization and from clinic A that a veterinary intern new to the practice was responsible for rabies vaccinations at the clinic when these animals were reportedly vaccinated. An internal review of this intern’s workstation revealed numerous rabies vaccine vials with higher than expected residual volume (i.e., used single-dose rabies vaccine vials were found still containing approximately 25%–33% of the original volume). This information, complemented by CDC's analysis, suggests that underdosing of rabies vaccine in dogs vaccinated at clinic A resulted in a higher than expected vaccination failure rate.

## Discussion

Ninety-nine percent of the estimated 59,000 human rabies deaths that occur worldwide annually are attributed to DMRVV (*2*). The United States eliminated DMRVV in 2007; therefore, the importation of an infected dog presents a risk for the reintroduction of DMRVV into the United States and risks the lives of persons and animals exposed during transit and rehoming. Public health responses to prevent the spread of DMRVV are costly to states: each DMRVV importation event is estimated to cost >$200,000, including public health response and health care costs (*3*).

During the last 15 years, five other instances of rabid dogs imported into the United States have been documented (*4*–*8*). This is the first instance during that time frame that resulted from vaccination failure rather than fraudulent or incomplete paperwork. OIE recommends that all countries verify adequate vaccination in dogs from countries with endemic rabies by requiring testing of antibody levels before entry. This OIE recommendation is not currently part of U.S. dog importation requirements. Although data about the outcomes of underdosing rabies vaccines in dogs are limited, large dogs might be more likely to have inadequate titers after rabies vaccination (*9*). The high vaccine failure rate detected in this investigation, with a higher likelihood of lower immune response to vaccination in larger dogs, is consistent with the expected immunologic response to low-dose vaccination.

On July 14, 2021, CDC temporarily suspended dog importations from high-risk DMRVV countries.^¶^ This suspension was implemented after CDC documented a substantial increase in attempts to import dogs with fraudulent or incomplete rabies vaccination certificates into the United States (*10*). As of this writing, the suspension is still in effect, and this investigation highlights the need for stronger controls for the importation of dogs from high-risk countries to prevent the reintroduction of DMRVV because the suspension is temporary and is not a long-term solution. A requirement for rabies vaccination certificates alone will not adequately identify improper vaccination practices or fraudulent paperwork and is insufficient as a stand-alone rabies importation prevention measure. Serologic testing of animals from high-risk countries and electronic reporting of results directly from prequalified laboratories before arrival in the United States should be considered to mitigate the risk of importing DMRVV.**

**Summary**

**What is already known about this topic?**

Since the elimination of dog-maintained rabies virus variants (DMRVV) from the United States in 2007, five rabid dogs have been imported into the United States from countries with high risk for DMRVV.

**What is added by this report?**

This is the first instance since 2007 that an importation of a rabid dog resulted from vaccination failure rather than fraudulent or incomplete paperwork. Serologic testing confirmed that 22% of 32 animals in this shipment from Azerbaijan were not adequately vaccinated before importation.

**What are the implications for public health practice?**

Rabies vaccination certificates alone will not ensure that dogs from high-risk countries for DMRVV have adequate protection against rabies. Serologic titers before importation would mitigate the risk for importing DMRVV.

## Footnotes

*
https://www.cdc.gov/importation/bringing-an-animal-into-the-united-states/high-risk.html

† https://www.cdc.gov/importation/bringing-an-animal-into-the-united-states/vaccine-certificate.html; https://www.govregs.com/regulations/expand/title42_chapterI_part71_subpartF_section71.51#regulation_1

§
https://www.cdc.gov/rabies/medical_care/index.html

¶
https://www.govinfo.gov/content/pkg/FR-2021-06-16/pdf/2021-12418.pdf

**
https://www.oie.int/en/what-we-do/standards/codes-and-manuals/terrestrial-code-online-access
